# DNA Methyltransferases, DNA Methylation, and Age-Associated Cognitive Function

**DOI:** 10.3390/ijms19051315

**Published:** 2018-04-28

**Authors:** Di Cui, Xiangru Xu

**Affiliations:** 1 Max Planck Institute for Biology of Ageing, 50931 Cologne, Germany; dcui@age.mpg.de; 2 Department of Anesthesiology, Yale University School of Medicine, New Haven, CT 06520, USA

**Keywords:** DNMTs, DNA methylation, synaptic gene expression, CNS, cognitive ageing

## Abstract

Ageing, a leading cause of the decline/deficits in human learning, memory, and cognitive abilities, is a major risk factor for age-associated neurodegenerative disorders such as Alzheimer’s disease. Emerging evidence suggests that epigenetics, an inheritable but reversible biochemical process, plays a crucial role in the pathogenesis of age-related neurological disorders. DNA methylation, the best-known epigenetic mark, has attracted most attention in this regard. DNA methyltransferases (DNMTs) are key enzymes in mediating the DNA methylation process, by which a methyl group is transferred, faithfully or anew, to genomic DNA sequences. Biologically, DNMTs are important for gene imprinting. Accumulating evidence suggests that DNMTs not only play critical roles, including gene imprinting and transcription regulation, in early development stages of the central nervous system (CNS), but also are indispensable in adult learning, memory, and cognition. Therefore, the impact of DNMTs and DNA methylation on age-associated cognitive functions and neurodegenerative diseases has emerged as a pivotal topic in the field. In this review, the effects of each DNMT on CNS development and healthy and pathological ageing are discussed.

## 1. DNA Methylation and DNA Methyltransferases

The term epigenetics was firstly coined by C. H. Waddington in 1942 to describe the interaction between genes and their surroundings [1]. It is a heritable change in gene expression regulation that differs from genetics: it does not involve any alterations of nucleotide sequences [2], and mainly includes DNA methylation, post-translational histone modifications, and non-coding RNA activity [3,4]. DNA methylation is the first and most widely studied epigenetic mark and plays important roles in regulating gene expression and maintaining genome stability in a spectrum of species including plants, rodents, and humans [5,6,7]. 

DNA methylation, in general, is defined as a biochemical process whereby s methyl group is added to DNA bases. It usually occurs on the fifth position of cytosine (C) to form 5-methyl cytosine, known as 5mC in the context of CpG dinucleotides—regions of DNA where a cytosine nucleotide is followed by a guanine nucleotide in the linear sequence of bases along its 5′→3′ direction in both eukaryotes and prokaryotes [5,6,7]. The effect of 5mC is traditionally considered to interfere with transcriptional initiation, thereby silences gene expression [8]. Other forms of DNA methylation exist, such as N6-adenine methylation (6mA), which was discovered lately in *Caenorhabditis elegans* and mammalian species as a new epigenetic mark [9,10] associated with gene transcriptional activation [4]. In this review, we will primarily focus on the cytosine DNA methylation process.

The DNA methylation process is mediated by DNA methyltransferase (DNMT). DNMT catalyzes the methylation of DNA elements by adding an extra methyl group (–CH3) which is donated by *S*-adenyl methionine (SAM), a universal methyl donor [11]. Three DNMTs have been identified in vertebrates, including DNMT1, DNMT3a, and DNMT3b, which are encoded by independent genes [12]. DNMTs are further divided into two functional categories, i.e., maintenance DNA methyltransferases (DNMT1) and de novo DNA methyltransferases (DNMT3a and DNMT3b).

**Figure 1:**
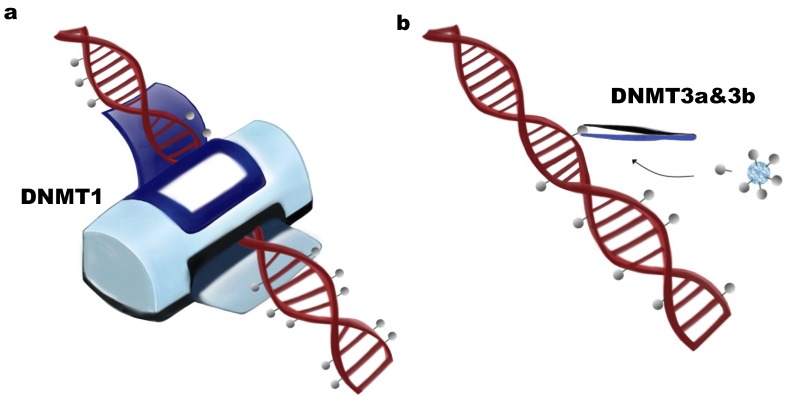
Biochemical functions of DNA methyltransferases. (**a**) DNMT1 has been defined as a maintenance DNA methyltransferase. The function of DNMT1 is to copy the existing methylation pattern, preferring hemi-methylated DNA; (**b**) De novo DNMTs include DNMT3a and DNMT3b, whose function is to establish new DNA methylation patterns by transferring methyl groups donated by *S*-adenyl methionine (SAM) to unmethylated DNA.

DNMT1 was the first cloned DNA methyltransferase [13] and is highly conserved between mouse and human [14]. DNMT1 contains 1620 amino acids, has a strong preference for hemimethylated DNA [15], and functions in maintaining the pre-existing DNA methylation patterns during DNA replication (Figure 1a). In contrast, DNMT3a and DNMT3b function mostly in establishing new DNA methylation patterns by catalyzing the methylation of unmethylated DNA elements in early embryonic development [6,16] (Figure 1b). One additional DNMT family member is DNMT3L (DNMT3-like), which is homologues to DNMT3a and DNMT3b, but lacks several residues and the catalytic domain. Although without enzyme activity, DNMT3L has been reported in vitro to function in the enhancement of DNA methylation activity of DNMT3a and DNMT3b, but not DNMT1, by direct interaction [17]. In vivo, DNMT3L can physically and functionally interact in the nucleus with DNMT3a2, an isoform of DNMT3, but not with DNMT3a or DNMT3b, inducing regional DNA methylation in mouse embryonic stem (ES) cells and mouse E16.5 embryos [18,19].

## 2. DNA Methylation, DNA Methyltransferases, and Mammalian CNS Development

DNA methylation is an essential dynamical biochemical process during the mammalian life cycle [20]. In the early embryonic developmental stages, DNA methylation decreases at a genome-wide scale, because of Ten-eleven translocation 3 (TET3)-dependent active demethylation and DNA replication-dependent passive demethylation [21,22]. From fertilization to cell cleavage, morula, and blastocyst, DNA methylation decreases, reaching the lowest coverage (43% in human and 10% in mouse) [23,24]. Starting from blastocyst formation, epigenetic reprogramming begins, and DNA methylation is re-established by DNMTs [25]. DNMTs level changes in different developmental stages in order to establish new methylation patterns and/or regulate cell/tissue differentiation and development.

DNA methylation has been reported to regulate neuronal differentiation in early CNS development. DNA methylation on the promoter of pluripotency and germline-specific genes determines pluripotency repression in progenitor cells, while, during terminal differentiation, neuron-specific genes are activated by switching off H3K27me3 on their promoters [26]. From mammalian fetal to adult brain development, a global methylome reconfiguration was found to be associated with synaptogenesis [27]. In humans, several months or years after birth, a dramatic rise of DNA methylation level is observed on 16/50 loci that are responsible for CNS growth and development, while the level changes dynamically in the cerebral cortex throughout the whole life, being involved in neuronal differentiation [28]. The analysis of the human prefrontal cortex from the fetal stage, through childhood (1–10 years of age), until post-childhood demonstrated that DNA methylation has an independent pattern in each age period. During the fetal stage, DNA methylation changes in both directions dramatically, with a rate of 80% per year. From the fetal to the postnatal period, 54.8% of overlapped CpG sites show an opposite pattern, a shift from decreased to increased methylation occurs with age [29], indicating that DNA methylation plays distinct roles in regulating CNS development in an age-related stage-dependent manner. Fascinatingly, the DNA methylation level has been identified lately as an epigenetic clock to represent the organismal biological ageing. In human, 353 CpG sites were characterized to form an epigenetic age clock [30]. In mouse, epigenetic predictors were also identified, using multiple tissues including brain, as a biological clock in certain age periods as well as to define the effectiveness of favorable age-related interventions [31,32,33]. 

**Figure 2:**
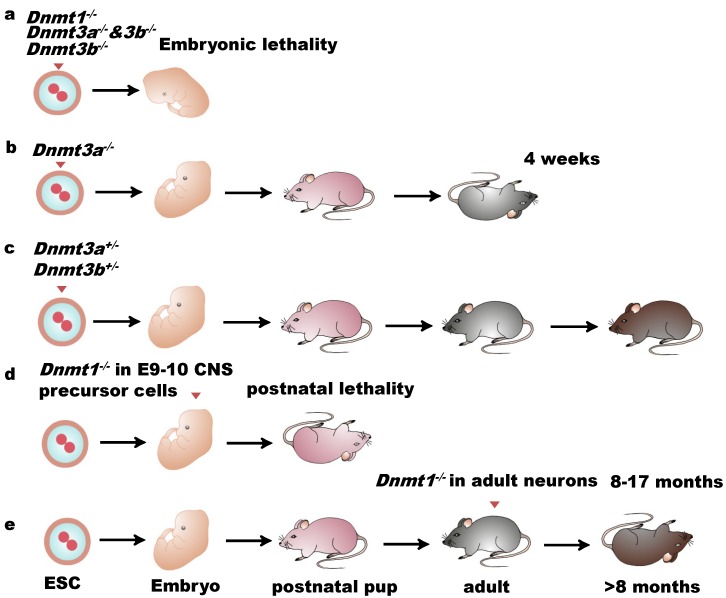
Biological functions of DNMTs in mammalian development. (**a**) Mutations in *Dnmt1* or *Dnmt3b* or loss of both *Dnmt3a* and *Dnmt3b* lead to embryonic or postnatal lethality; (**b**) *Dnmt3a*-null mice can survive up to four weeks; (**c**) *Dnmt3a-* or *Dnmt3b*-heterozygous-deficient mice perform normally; (**d**) Embryos with *Dnmt1*-deficient CNS precursor cells starting from E9–E10 died in the postnatal period; (**e**) *Dnmt1* deletion in neurons of adult mice allows mice survival for 8 to 17 months.

DNMTs are abundant in the embryonic stage and significantly decrease after the terminal differentiation stage. Studies have been conducted on the mutations of DNMTs in order to investigate the functions of DNA methylation in brain development. Mutations in *DNMT1* or *DNMT3a/3b* genes in mouse germlines block development and cause lethality of the embryos [34,35] (Figure 2a–c), suggesting that DNMTs play crucial roles in embryogenesis. The importance of DNA methylation in early development, embryonic stem cells, and adult lineages in many aspects has been carefully reviewed [36]. We, here, mainly focus on the function of DNMTs in the central nervous system (CNS).

*DNMT1* was detected as highly expressed in the CNS during embryonic stages and in postnatal mice, not only in dividing cells but also in neural precursor cells and postmitotic neurons [37,38]. Loss of *DNMT1* in the dorsal forebrain results in somatosensory barrel cortex failure [39], revealing an essential role of DNMT1 in the development of sensory maps and synaptic plasticity. Conditional deletion of DNMT1 in neuroblasts of E12 embryos affects CNS neurons in hypomethylation and viability [40] (Figure 2d). *In vitro*, cerebellar neurons with *DNMT1* deletion survived well for two weeks without decreasing their global DNA methylation, indicating that DNMT1 is not indispensable for neuronal survival and for maintaining the global methylation level. *In vivo*, embryos with *DNMT1*-deficient CNS precursor cells from E9–E10 died in the postnatal period (Figure 2d). However, when *DNMT1* is deleted in the neurons of adult mice, the animals can survive for 8 to 17 months [40] (Figure 2e), suggesting that DNMT1 is crucial for neurogenesis, while is not essential for mature neuronal survival. Recently, disruption of *DNMT1* together with *DNMT3a* and *DNMT3b* has been reported to affect mammalian retina development [41]. In human, a *DNMT1* mutation was found in patients affected by HSANIE, a hearing loss disorder of the CNS and peripheral nervous system [42,43,44].

The expression pattern of DNMT3a and DNMT3b has been examined in mammalian developing embryos and adult tissues [45,46]. High expression of DNMT3a and DNMT3b has been detected in ES cells as well as embryos. In late-stage embryos, DNMT3a becomes ubiquitously expressed, while DNMT3b expression level decreases but remains high in the brain [34], revealing their overlapping functions in CNS development. DNMT3a2, the shorter isoform of DNMT3a, is hardly detectable in most somatic tissues, but can be highly detected in ES cells and germ cells as well as in the adult cortex [47,48], suggesting that DNMT3a2 plays a key role in early de novo methylation establishment and brain function. The Loss of both DNMT3a and DNMT3b in mouse leads to embryonic or postnatal lethality [47], without affecting the maintenance of the imprinted methylation patterns [34]. DNMT3a or DNMT3b-heterozygous-deficient mice perform normally, while DNMT3a-null mice have development disorders and can only survive up to four weeks, and DNMT3b-null mice are not viable [34], indicating their essential role in early development. In humans, around 20% of AML (acute myeloid leukemia) and MDS (myelodysplastic syndromes) patients carrying mutations in DNMT3A suffer from the loss of over 50% of DNA methylation [49]. DNMT3B heterozygous mutation results in an autoimmune syndrome called ICF (immunodeficiency, centromeric instability, and facial anomalies). Aberrant gene expression in the neurogenesis in ICF patients was reported [50,51,52], and an ICF mouse model was generated by disrupting DNMT3b [53].

## 3. DNA Methylation, DNA Methyltransferases, Mammalian CNS Ageing, and Alzheimer´s Disease 

Ageing is associated with a decline of the cognitive function. Cognitive decline in ageing basically includes attention and memory deficits, among which working memory is impaired most significantly in old individuals [54]. The concept of working memory is not a unitary cognitive function but was coined as a cognitive system that processes and stores information, such as learning, cognition, and memory [55,56]. Brain frontal regions have been demonstrated to be involved in working memory. In particular, the prefrontal cortex has been identified as functioning in spatial working memory, by positron emission tomography (PET) and functional magnetic resonance imaging (fMRI) [57,58]. Loss or damage of hippocampal tissues disrupts episodic and spatial memories [59,60]. Additionally, the neural networks in the hippocampus and prefrontal cortex have been reported to play a key role in spatial working memory and ageing [59], revealing that the frontal cortex and hippocampus are main brain regions involved in working memory. How cognitive functions are mediated by brain regions, especially the hippocampus will be discussed further. 

**Figure 3:**
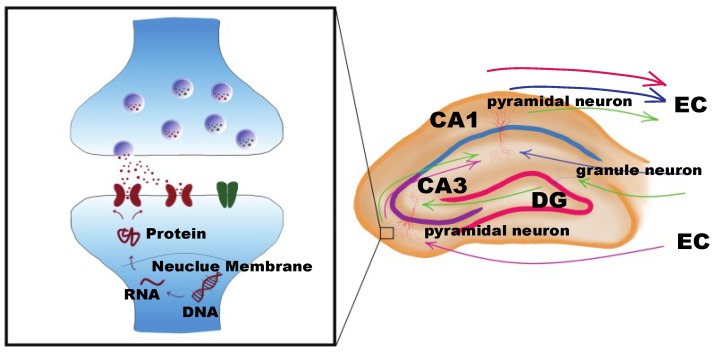
Hippocampal circuits and synaptic plasticity. Information is delivered from the entorhinal cortex (EC) to the dentate gyrus (DG) region by granule cell mossy fibers; (cornu ammonis) CA3 receives inputs from the mossy fibers and projects to CA1 via the Schaffer collateral fibers of pyramidal neurons. CA1 passes the information to the EC (circuit in green). In the circuits, CA3 (circuit in pink) or CA1 (circuit in blue) also can receive information directly from EC. Information spreads through the synapses between neurons. DNA methylation regulates RNA transcription of synaptic plasticity genes, which can modulate ion channel receptors, in turn mediating synaptic plasticity in circuits.

Both learning and memory represent neuronal activities accomplished predominantly by proper hippocampus function. Adult hippocampal neurogenesis is considered to contribute to higher cognitive functions, and new neurons are produced by one of the two neurogenic zones of the brain in the hippocampus, i.e., the dentate gyrus (DG) of hippocampus. [61,62,63]. Furthermore, the hippocampus is the center where primary cell groups form neuronal circuits connecting with other brain regions [6,64]. The hippocampus contains three fields, i.e., cornu ammonis 1 (CA1), cornu ammonis 3 (CA3), and DG (Figure 3), which are reported to be particularly important for memory formation and are susceptible to stress and glucocorticoids and age-associated learning deficits, respectively [65,66]. The mammalian brain stores memory through neuronal circuits that are formed by synaptic connections between individual neurons. Specifically, information is delivered from the entorhinal cortex to the DG region by granule cell mossy fibers. CA3 receives inputs from the mossy fibers as the main extrinsic projection of DG and projects to CA1 via the Schaffer collateral fibers. The hippocampal circuit is closed when CA1 passes the information to the entorhinal cortex [66] (Figure 3). In the ageing process, memory impairments can be caused by the reduction of synaptic connections, such as axosomatic synapses in DG or hippocampal excitatory synapses in CA1 [67,68]. 

The DNA methylation levels change with normal ageing in various tissues, including the brain. Early studies reported that, in both human and rodents, ageing is correlated with a global decrease in DNA methylation, which varies depending on the site [3,69,70,71]. Gene-specific DNA methylation changes are involved in rewarding in a context-dependent manner and is essential for memory formation, neurogenesis, and neuronal plasticity [72,73]. Disrupting DNA methylation by using DNMT inhibitors in the ventral tegmental area, where rewarding-related dopamine neurons are located, reduced significantly the rewarding response. [72]. During memory formation, DNA methylation regulates genes in a dynamic way over time, maintaining contextual fear conditioning rote memory. Hypermethylation on memory positive regulator genes like *Reln* (reelin) was detected after one hour of training, while, for memory repressors like *CaN* (calcineurin), methylation started to increase one day later. Disrupting cortical DNA methylation interfered with *CaN* transcription after 30 days of training, in turn inhibiting remote memory recall [74]. In mature CNS, neuronal plasticity and long-term memory have been shown to be modulated by DNA methylation through DNMTs activity in the hippocampus [75]. The neuronal methylome changes dramatically after neuronal activity, associating with synaptic plasticity genes gaining or losing DNA methylation [76] (Figure 3). Additionally, adult neurogenesis is defined as a pivotal process in the generation of neurons in adulthood and thus directly affects learning and memory functions [77]. It has been pointed out that hypomethylation in the brain during aging is responsible for declined adult neurogenesis [78]. 

As DNMTs mediate the DNA methylation process, the expression levels of DNMTs were also found to decrease with age. Specifically, in both human and mouse brains, compared to young age, our preliminary study indicates that DNMT1, DNMT3a, and DNMT3a2 levels are decreased in both frontal cortex and hippocampal tissues (Xu et al., unpublished data). Moreover, DNMTs have been closely linked to memory and cognitive functions [79], suggesting that DNA methylation is a critical mechanism in regulating age-associated cognition. 

DNMT1 level in the brain is higher than in other tissues but varies among different brain regions [78]. Some key genes that are involved in neuronal growth, differentiation. or synaptic plasticity are regulated. For example, the gene coding for brain-derived neurotrophic factor (*Bdnf*)—a secreted neurotrophin—has been firmly implicated in neuronal differentiation and survival and has also emerged as a vital regulator of synaptogenesis and synaptic plasticity mechanisms underlying learning and memory in adults [80]. In primary cultured neurons, the expression of *Bdnf* is significantly increased when Dnmt1 is mutated, indicating that DnmtT1 reversibly modulates *Bdnf*. However, Dnmt1 inhibition in mice causes fear memory deficits [81]. Conditional double mutation of Dnmt1 and Dnmt3a in mouse cortical neurons results in learning and memory impairments, but this is not observed in Dnmt1 or Dnmt3a single-knock-out animals [82]. Collectively, a dynamic level of Dnmt1 mediates learning and memory function, at least partially through synaptic plasticity genes regulation in adult CNS.

The expression of DNMT3a, as a *de novo* DNA methyltransferase, remains high in adult neurons. *Dnmt3a*-deficient mice did not present an impairment in long-term memory or learning, which was observed in *Dnmt3a* and *Dnmt1* double knock-out mice, indicating that Dnmt1 can partially substitute Dnmt3a in synaptic plasticity, learning, and memory [82]. The role of Dnmt3a in cognitive function is specific in episodic memory and cannot be compensated by Dnmt1 [83]. A mild cognitive impairment was reported to be associated with Dnmt3a, but it is still debatable as other report showed negative results about this correlation [84,85]. Dnmt3a mediates cognitive functions in associative memory, while it appears not to have an indispensable role in synaptic plasticity and learning. Dnmt3a2, however, is largely associated with cognitive function in a positive manner. Transient knockdown of Dnmt3a2 in young mice hippocampal tissues accelerates cognitive ageing, while overexpression of Dnmt3a2 has a rescue effect on memory in old mice. Furthermore, an enhancement of young adult memory was observed following Dnmt3a2 overexpression in the hippocampus [79,86], revealing that the expression level of Dnmt3a2 functionally correlates with age and positively regulates age-associated learning and memory in both young and old individuals, while the molecular mechanism still remains largely unknown. 

Dnmt3b and Dnmt3a mRNA were demonstrated to increase after fear conditioning [87]. A study of monozygotic twins in a human cohort indicates that DNMT3B is correlated with cognitive ability in response to environment cues [88], suggesting that DNMT3b is linked with neurocognitive function dynamically in an environment-dependent manner. 

Alzheimer´s disease (AD) is one of the most devastating age-related neurodegenerative diseases. The typical symptoms of AD are difficulties in language, memory loss, and personality changes. AD has become a worldwide concern, as over 17% of 65-year-old people, and over 50% of 85-year-old people suffer from AD [89,90]. Genetically, it has been demonstrated that mutations in amyloid protein precursor (APP), presenilin 1 (PSEN1), and presenilin 2 (PSEN2) contribute to familial early onset AD, referring to the families that are affected by AD earlier than 65th year of age [91,92]. However, the underlying mechanism for late-onset AD is still not clear, and no gene has been found yet to be responsible. Recently, epigenetic factors have been taken into the consideration, including DNA methylation. 

An epigenetic regulatory mechanism has been reported to contribute to AD, in which lower levels of DNA methylation on the promoter of target genes were detected [93]. The expression of DNMT1 and global 5mC and 5hmC were shown to be decreased in AD entorhinal cortex island neurons and hippocampus [94,95]. Similarly, in peripheral blood samples of late-onset AD patients, a decreased methylation was detected in the mitochondrial DNA displacement loop region, which is reported to be involved in pivotal processes like DNA repair. Besides, a human study showed that the change of DNA methylation on certain genes loci is possibly connected to AD [96]. Patients with amnestic mild cognitive impairment develop AD associated with an increase of DNA methylation on *Bdnf* promoter, which can be responsible for the reduction of mRNA or protein levels of BDNF [97], indicating that a higher methylation is associated with a lower mRNA expression. In contrast, Triggering receptor expressed on myeloid cells 2 (TREM2), is an immune receptor whose expression indicates a higher risk for AD [98,99]. The mRNA level of TREM2 is significantly higher in AD patients, however, it shows a lower DNA methylation level at intron 1 [100]. Additionally, hypomethylation in certain genes in a tissue-specific way was detected in AD brains or in patients suffering from other neurodegeneration diseases [101,102], indicating a correlation between DNA methylation and AD, which varies with respect to DNA elements/regions or tissues. This suggests that DNA methylation is probably involved in AD pathogenesis, through gene promoter or intron methylation, though the molecular mechanisms still remains largely unexplored. 

## 4. Mechanisms of DNA Methyltransferases in Regulating Neuronal Synaptic Plasticity-Related Gene Transcription

Age-associated cognitive deficits are closely linked to the dysfunction of neuronal synaptic plasticity. Neuronal synaptic plasticity, however, is regulated by a family of immediate-early expressed genes, for example, activity-regulated cytoskeleton-associated protein (Arc). The mRNA of Arc is required to activate *N*-methyl-d-aspartate (NMDA) receptor in synapses, which is critical in controlling synaptic plasticity [103,104]. Loss of Arc in mice causes memory impairments [105]. However, the underlying molecular mechanisms in regulating gene expression modulating synaptic plasticity that strengthens cognitive functions are largely unclear. Accumulating studies demonstrate that changes in the DNA methylation level of plasticity-relevant genes is pivotal for learning and memory [106,107,108,109]. In terms of the mechanisms regarding DNMT-regulated synaptic plasticity gene expression, so far mainly two potential mechanisms are known. 

### 4.1. DNMTs Regulate Neuronal Gene Transcription by Fine-Tuning the Methylation Pattern on DNA Elements Such as Promoter, Enhancer, and Gene Body

#### 4.1.1. DNMTs Inhibit Gene Transcription

**Figure 4:**
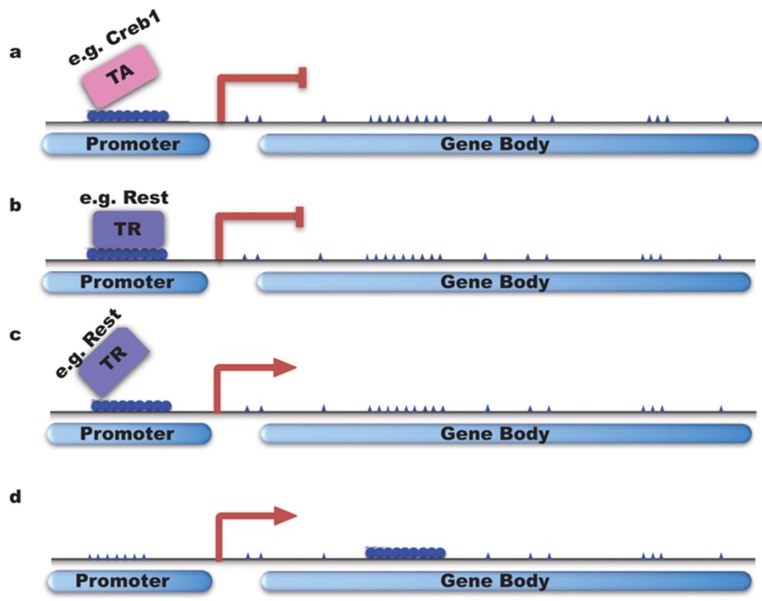
DNA methylation and neuronal gene transcription. (**a**,**b**) DNA methylation can inhibit gene transcription by preventing the loading of TA (transcription activator e.g., Creb1) or facilitating transcription repressor binding; (**c**,**d**) DNMTs can activate gene transcription by inhibiting the binding of TR (transcriptional repressor e.g., Rest) or by gene body methylation.

DNA methylation occurs in preference on CpG sites, where a guanine nucleotide follows a cytosine. In the mammalian genome, 70% of CpG sites are methylated [110], while CpG islands (CGIs), which are short DNA sequences rich in CpG sites and always associated with transcriptional starting sites, are mostly unmethylated. More than 70% of promoters contain CGIs [111,112]. In mammals, the methylation of CGIs near transcriptional starting sites is related to long-term gene silencing [113], revealing that normally methylated promoters are associated with gene silencing. For a long time, DNA methylation has been a correlated with gene transcription silencing. Studies have shown that low transcription is linked to DNA methylation near gene promoter regions. For example, DNMT3a decrease was reported to correlate with an increase of Arc expression, possibly due to lower methylation on Arc promoter [114]. In this case, gene silencing is believed to work largely by preventing the loading of transcription factors (Figure 4a), which will inhibit gene transcription [6,115], or by facilitating the binding of transcription repressors. For example, DNMT3b can methylate non-CpG sites which enhances the binding of neuron-restrictive silencer factor/RE1-silencing transcription factor (NRSF/REST) (Figure 4b), which is a neuronal gene negative regulator [116]. Gene transcription is also reported to be regulated by gene enhancer methylation in a methylation-degree-dependent manner. For instance, hypermethylation and hypomethylation of enhancers of cancer genes are correlated with gene up- or downregulation in cancer cells, respectively [117]. 

#### 4.1.2. DNMTs Activate/Enhance Gene Transcription

On the other hand, DNA methylation in some cases can also trigger transcription by inhibiting the binding of transcriptional repressors [6,118] (Figure 4c). Studies on the binding profiling of Rest showed that all sequences with gain of DNA methylation prevent Rest binding during neural progenitors differentiation [119]. Recently, methylation on the gene bodies of active genes was discovered (Figure 4d), and the relationships between methylation of CGIs on gene body regions and transcription are different depending on tissue or cell types [120]. For instance, a higher methylation level on gene body regions induced by DNMT3B was correlated with active genes in human tissues [121]. The mechanism for such a correlation remains unclear.

### 4.2. DNMTs Regulate Neuronal Gene Transcription by Coordinating the Function Of Methyl-DNA-Binding Proteins and Histones

#### 4.2.1. DNMTs Associates with Methyl-DNA-Binding Protein in Neuronal Gene Regulation

DNA methylation can recruit proteins with a methyl-CpG binding domain (MBD), which was first isolated from methyl CpG binding protein 2 (MeCP2) [122]. MeCP2 is a typical DNA methylation binding protein, which has been shown to regulate neuronal function, mainly inducing gene repression. The DNA methylation pattern mediated by DNMT3a was recently demonstrated to be associated with MeCp2 and gene expression in early neuronal development [123]. Previously, DNMT1 was reported to interact with MeCP2 largely depending on the transcription repressor domain (TRD) of MeCP2 [124]. The TRD domain is connected with various repressor machineries, such as histone deacetylase (HDAC), which could block gene transcription [125,126]. Besides the MBDs family, other families are known, such as the ubiquitin-like protein (UHRF) family, which is reported to functionally target DNMT1 and DNMT3a [127,128] and affect neuronal differentiation and survival [129].

#### 4.2.2. DNMTs Associate with Histone Modification in Neuronal Gene Regulation

As mentioned before, HDAC1 can be recruited by MeCP2, thereby binds with DNMT1. Similarly, a previous study elucidated a more direct way of DNMT1 in regulating HDAC1 activity, consisting in the interaction with histone deacetylase, trithorax-related proteins (HRX or MLL) [130]. Neuronal function can be mediated by different histone modifications. HDAC2 negatively regulates synaptic plasticity and learning, while HDAC2 induces cognitive enhancement [131]. By recruiting different histones, DNMTs are able to regulate neuronal gene transcription, however to what extent DNA methylation, histone modifications, and other epigenetic factors, like non-coding RNAs, functionally overlap is not clear.

## 5. Perspectives of DNA Methylation on Cognitive Ageing

Apart from genetics, epigenetics has become the second large focus in studies on healthy cognitive ageing and age-related diseases. As the most studied epigenetic mark, DNA methylation has a pivotal role in regulating genes involved in these processes. Firstly, DNA methylation mediates early CNS development, being involved in neuron growth, neuron differentiation, and adult neurogenesis. Thus, DNMTs are dispensable in regulating the genes that actively participate in the cognitive processes of learning and memory, which are turned off in early development to allow differentiation and subsequent influence the structural development and organogenesis that are required for abstract learning and memory functions during the life-time of both species. However, premature expression of this panel of genes could rather interfere with proper structural development and, therefore, their expression is inhibited by development-specific methylation that gradually ceases in a tissue/region-specific manner later in life, thereby eliminating methylation and initiating localized expression. The functions of DNA methylation and DNMTs were mostly investigated by genetic mutations in distinct age stages, and targeted sites have been detected associated with brain development. 

Aging is a gradual and progressive decline in physiological integrity with specific and diverse hallmarks of molecular and cellular processes [132]. Accelerated aging due to single-nucleotide changes that abolish splice sites (Progeria) and mutated genome (Huntington’s) indicate a molecular interplay that modulates the epigenome quantitatively and qualitatively. One of the prime aging molecules, Progerin, is detected very early (around 3 years of age) but it rapidly accumulates leading to Progeria. Progeric children have cardiac defects similar to those in people of 70 years of age; nevertheless, their brain and learning ability is completely normal and age-appropriate, indicating tissue-specific differential aging: the learning and memory functions of the brain appear to be resistant to the accelerated aging of heart and alopecia in Progeric individuals [133]. Enteric neurons display higher vulnerability to cell death and degeneration than neurons in other parts of the nervous system [134]. Given the fact that DNMTs are more abundant in the CNS than in any other tissues, including heart, stomach, and intestines, these studies suggested a great role of such molecules in defying brain aging at the epigenetic level.

Accumulating researches have elucidated that DNA methylation is associated with learning, memory, cognition, and AD. Meanwhile, DNA methylation patterns have been identified as an epigenetic age clock in different tissues, including human and mouse brains. The link between DNA methylation and ageing has been widely accepted; however, its molecular mechanisms still remain largely unclear. Some studies indicate a possible model of how DNA methylation regulates gene expression related to age-associated cognitive ageing or AD pathology. Currently, DNA methylation in mediating brain ageing has been reported to highly vary depending on the gene and on the tissue. Moreover, DNMTs family, which mediates the process of DNA methylation, is reported to be responsible of memory formation or cognitive deficits. However, DNA methylation in the brain, especially during old age, need to be further investigated, to understand the DNA methylation pattern of different types of neurons in memory or locomotion and how DNA methylation directly or indirectly targets genes that are responsible for synaptic plasticity. Besides, the patterns of DNA methylation and other epigenetic marks like histone modification need to be further addressed to understand their role in the cognitive ageing process.
